# Misinformation about COVID-19: evidence for differential latent profiles and a strong association with trust in science

**DOI:** 10.1186/s12889-020-10103-x

**Published:** 2021-01-07

**Authors:** Jon Agley, Yunyu Xiao

**Affiliations:** 1 Prevention Insights, School of Public Health, Indiana University Bloomington, 809 E. 9th St., Bloomington, IN 47405 USA; 2 Department of Applied Health Science, School of Public Health, Indiana University Bloomington, 809 E. 9th St., Bloomington, IN 47405 USA; 3 School of Social Work, Indiana University-Purdue University Indianapolis (IUPUI), Indianapolis, IN USA; 4 School of Social Work, Indiana University Bloomington, Bloomington, IN USA

**Keywords:** COVID-19, Misinformation, Trust, Conspiracy theories, Coronavirus

## Abstract

**Background:**

The global spread of coronavirus disease 2019 (COVID-19) has been mirrored by diffusion of misinformation and conspiracy theories about its origins (such as 5G cellular networks) and the motivations of preventive measures like vaccination, social distancing, and face masks (for example, as a political ploy). These beliefs have resulted in substantive, negative real-world outcomes but remain largely unstudied.

**Methods:**

This was a cross-sectional, online survey (*n*=660). Participants were asked about the believability of five selected COVID-19 narratives, their political orientation, their religious commitment, and their trust in science (a 21-item scale), along with sociodemographic items. Data were assessed descriptively, then latent profile analysis was used to identify subgroups with similar believability profiles. Bivariate (ANOVA) analyses were run, then multivariable, multivariate logistic regression was used to identify factors associated with membership in specific COVID-19 narrative believability profiles.

**Results:**

For the full sample, believability of the narratives varied, from a low of 1.94 (SD=1.72) for the 5G narrative to a high of 5.56 (SD=1.64) for the zoonotic (scientific consensus) narrative. Four distinct belief profiles emerged, with the preponderance (70%) of the sample falling into Profile 1, which believed the scientifically accepted narrative (zoonotic origin) but not the misinformed or conspiratorial narratives. Other profiles did not disbelieve the zoonotic explanation, but rather believed additional misinformation to varying degrees. Controlling for sociodemographics, political orientation and religious commitment were marginally, and typically non-significantly, associated with COVID-19 belief profile membership. However, trust in science was a strong, significant predictor of profile membership, with lower trust being substantively associated with belonging to Profiles 2 through 4.

**Conclusions:**

Belief in misinformation or conspiratorial narratives may not be mutually exclusive from belief in the narrative reflecting scientific consensus; that is, profiles were distinguished not by belief in the zoonotic narrative, but rather by concomitant belief or disbelief in additional narratives. Additional, renewed dissemination of scientifically accepted narratives may not attenuate belief in misinformation. However, prophylaxis of COVID-19 misinformation might be achieved by taking concrete steps to improve trust in science and scientists, such as building understanding of the scientific process and supporting open science initiatives.

**Supplementary Information:**

The online version contains supplementary material available at 10.1186/s12889-020-10103-x.

## Background

As coronavirus disease 2019 (COVID-19) has spread around the globe, the scientific community has responded by conducting and providing unprecedented access to research studies related to COVID-19 [1]. Early in the course of the pandemic, researchers noticed the spread of misinformation, conspiracy theories (causal attribution to “machinations of powerful people who attempt to conceal their role”) [2], and unverified information about COVID-19 [3, 4], which has taken the form of false/fabricated content and true information presented in misleading ways [5]. This deluge of information has introduced confusion among the public in terms which sources of information are trustworthy [6], despite the open conduct of epidemiological research and other scientific work on COVID-19.

Although one might expect that improved access and visibility of research would result in increased trust being placed in scientists and the scientific enterprise, a preliminary study failed to find such a change between December 2019 and March 2020 in the United States (US) [7]. Peer reviewed studies exist alongside misinformation about medical topics, the latter of which is easily accessible in the US and is associated with differential health behaviors (e.g., who gets a vaccine, or who takes herbal supplements) [8]. As we describe and demonstrate subsequently, belief in misleading narratives about COVID-19 can have substantive, real-world consequences that makes this both an important theoretical and practical area of study. At the same time, evidence suggests that belief in misinformation is not pathological, but rather that it merits treatment as a serious area of scientific inquiry [9].

### Misinformation and conspiracy theories

Research on misinformation and conspiratorial thinking has burgeoned in recent years. Because this work has focused both on misinformation and conspiratorial thinking, we use these terms consistently with the specific studies cited, but somewhat interchangeably.

Consistent with the proliferation of misinformation about COVID-19, it has been proposed that conspiratorial thinking is more likely to emerge during times of societal crisis [10] and may stem from heuristic reasoning (e.g., “a major event must have a major cause”) [11]. At the same time, endorsement of misinformation or conspiracy seems to be common, with evidence from nationally representative research indicating that approximately half of US residents endorsed at least one conspiracy in surveys from 2006 to 2011, even when only offered a short list of possibilities [12]. A recent study of COVID-19 conspiracy theories similarly found that nearly 85% of a representative US sample of 3019 individuals believed that at least one COVID-19 conspiracy theory was “probably” or “definitely” true [13]. The widespread nature of this phenomenon logically suggests that endorsing misinformation is unlikely to be caused by delusions or discrete pathology.

#### Factors associated with beliefs

Previous research on factors associated with belief in misinformation or conspiracy theories has produced varying, and sometimes inconsistent, findings. The endorsement of misinformation has been found to vary across sociodemographic groups. For example, studies have identified that both low [14] and high [15] education levels are positively associated with belief in certain conspiratorial ideas. In addition, individuals who perceive themselves to be contextually low-status may be more likely to endorse conspiracy theories, especially about high-status groups, but social dynamics likely affect this substantively [16].

Political orientation is generally believed to be associated with conspiratorial endorsement or belief in misinformation, and some studies have reported that conservatism predicts believing or sharing misinformed narratives. For example, sharing “fake news” on Facebook during the 2016 US presidential election was associated with political conservatism and being age 65 or older, though researchers acknowledged potential omitted variable bias and pointed to the potential confounding (unmeasured) role of digital media literacy [17]. However, other researchers have suggested that strong political ideology on either side (left *or* right) is more explanatory [18], and that associations vary depending on the political orientation of the conspiracy or misinformation itself [19]. Consistent with the latter explanations, a preprint by Pennycook et al. examined data from the US, Canada, and the UK and found that cognitive sophistication (e.g., analytic thinking, basic science knowledge) was a superior predictor of endorsing misinformation about COVID-19 than political ideology, though none of the included variables predicted behavior change intentions [20]. This mirrored his prior finding that lower levels of analytic thinking were associated with inability to differentiate between real and fake news [21].

Though less well studied, religiosity, too, may be associated with general conspiratorial thinking (e.g., believing that “an official version of events could be an attempt to hide the truth from the public”), but the relationship is likely complex and mediated by trust in political institutions [22]. Researchers have also posited positive, indirect relationships between religion and endorsement of conspiracy theories. This might have a basis in the conceptual similarity between an all-powerful being (as described in many religions) and a hidden power orchestrating events or hiding the truth, the latter of which is a core feature of conspiratorial thinking [15].

### The importance of misinformation about COVID-19

Misinformation about COVID-19 is an important area of study not just theoretically, but also because of the potential for these beliefs to lead to real-world consequences. The present study examined four core misperceptions about COVID-19that contributed to short-term adverse consequences (situated alongside a fifth narrative that reflects scientific consensus). The misperceptions were drawn from Cornell University’s Alliance for Science, which prepared a list of current COVID-19 conspiracy theories in April, 2020 [23]. These were:

- [5G Narrative] Although viruses cannot be spread through wireless technology, theories associating 5G wireless technology with COVID-19 have proliferated [24] and led to more than 70 cell towers being burned in Europe (predominantly the United Kingdom) and Canada [25].
- [Gates Vaccine Narrative] Between February and April 2020, varied conspiracies linking Bill Gates to COVID-19 (e.g., as a pretext to embed microchips in large portions of the global population through vaccination) were the most ubiquitous of all conspiracy theories related to the virus [26]. Among other direct consequences, a non-government organization that became linked with this theory ended up calling the US Federal Bureau of Investigation for help after being targeted online [27].
- [Laboratory Development Narrative] Research indicates that COVID-19 is a zoonotic virus (see papers published in February by Chinese scientists [28] and in March by a group of scientists from the US, United Kingdom, and Australia [29]). However, officials in both the US and China have accused the other country of purposefully developing COVID-19 in a laboratory, often with the implication of military involvement [30, 31].
- [Liberty Restriction Narratives] While less clear-cut than the other examples provided here, debate has continued as to the seriousness of COVID-19 and the appropriate set of public health responses. A common thread has been the assertion that the true threat from COVID-19 relates to liberty (e.g., mask requirements, social distancing) rather than the virus itself. In some cases, individuals who have publicly derided proposed protective measures like social distancing have subsequently died from COVID-19 [32]. In other cases, these disagreements have become vitriolic and couched as a deliberate infringement on Constitutional rights. For example, the Governor of Kentucky was hung in effigy during a protest during Memorial Day weekend [33], and there has been a series of incidents where preventive measures like mask-wearing in public have become brief, violet flashpoints, resulting in outcomes up to and including murder [34].

We cannot be certain, yet, about the long-term effects of beliefs about COVID-19on the landscape of US politics, treatment of vulnerable populations, and other longer-term outcomes. Lessons from prior viral epidemics such as human immunodeficiency virus (HIV) suggest that misinformation like AIDS denialism, when embedded, can result in avoidable morbidity and mortality [35]. Further, in the time between preparation and submission of this article (May/June 2020) and revision during peer review (October 2020), researchers have also begun to strongly suggest the need for continued and multifaceted research on COVID-19 misinformation, including the nature of misinformed beliefs and how to prevent their uptake. For example, the editorial board of *The Lancet Infectious Diseases* issued a warning about the impact of COVID-19 misinformation in August [36]. Dr. Zucker, the Health Commissioner for New York State, published a commentary indicating that combatting online misinformation is “a critical component of effective public health response” [37]. Other concerning outcomes have also begun to manifest. Perhaps most notably, the U.S. Federal Bureau of Investigation prevented an attempted kidnapping and overthrow of the Governor of the State of Michigan in early October 2020 that was predicated, at least in part, by the perception that a statewide mask mandate for COVID-19 was unconstitutional [38]. Coincidentally, an interrupted time-series study published the same week illustrated the efficacy of non-pharmaceutical preventive behaviors, such as mask use, on reducing morbidity and mortality from COVID-19 [39]. Clearly, research on COVID-19 misinformation has both a theoretical and practical underpinning.

### Addressing misinformation

Misinformation can be difficult to address in the public sphere because it requires the source of information be trusted [40], while the very nature of misinformation often hypothesizes that experts or authorities are working to conceal the truth. Krause and colleagues (2020) note that it is important for scholars to be honest and transparent about the limits of knowledge (e.g., uncertainty), and that simply asserting one’s trustworthiness or accuracy is likely an insufficient step to take [40]. Further, one cannot assume that “fact checkers” are trusted by the public to be objective, or that objective presentation of data will simply overturn misinformation, especially when it is value-laden [40]. Timing of information provision may also matter. Studies have suggested that people may be less inclined to share or endorse misinformation or conspiracy theories if they are presented with reasoned, factual explanations prior to their exposure to misinformation [41]. However, this was not found to be true after exposure; stated differently, factual information may be capable of prevention, but not treatment [41]. This finding is consistent with theories about fact-based inoculation conferring resistance to argumentative persuasion [42].

Adding additional complication, just as misinformation tends to proliferate within a social echo chamber where few individuals interact with content “debunking” misinformation, scientific information tends to be shared within its own echo chamber. Thus, it may be rarely interacted with by those who do not already agree with the content [43]. So even if a scientific source of information is trusted, and “gets out ahead” of misinformation, there is a risk it will never reach its intended audience. The summed total of this information led us to conclude that: (a) it is both practically and theoretically important to understand the factors underlying endorsement of misinformation about COVID-19, (b) certain indicators might be, but are not definitively, associated with endorsement of misinformation, including political orientation, religious commitment, and education level, and (c) if scientists and “fact checkers” are not trusted by some individuals (whether rightly or wrongly), the degree of trustworthiness assigned to scientists may be an underlying mechanism that can explain belief in conspiratorial theories about COVID-19.

To investigate this question, we adopted a person-centered approach to identify profiles of beliefs about COVID-19 narratives. Importantly, these profiles incorporated perceived believability not only of misinformation, but also of a scientifically-accepted statement about the zoonotic source of COVID-19. To identify belief profiles, we used Latent Profile Analysis (LPA), a specific case of a finite mixture model that enables identification of subgroups of people according to patterns of relationships among selected continuous variables (i.e., “indicators,” in mixture modelling terminology) [44]. The goal of LPA is to identify the fewest number of latent classes (i.e., homogenous groups of individuals) that adequately explains the unobserved heterogeneity of the relationships between indicators within a population.

We hypothesized that 1) there are distinct profiles of individuals’ beliefs in different narratives related to COVID-19; 2) trust in science and scientists, as conceptualized in prior research on this topic [7, 45], is lower among subgroups that endorse misinformation or conspiracy theories about COVID-19, even after controlling individuals’ sociodemographic characteristics, political orientation and religious commitment.

## Methods

### Data collection

Data were obtained on May 22, 2020, from a sample of 660 US-based Amazon Mechanical Turk (mTurk) users ages 18 and older (individuals must be age 18 or older to enroll as an mTurk worker). A relatively new data collection platform, mTurk allows for rapid, inexpensive data collection mirroring quality that has been observed through traditional data collection methods [46, 47], including generally high reliability and validity [48]. Though not a mechanism for probability sampling [48], mTurk samples appear to mirror the US population in terms of intellectual ability [49] and most, but not all, sociodemographic characteristics [50].

To ensure data quality, minimum qualifications were specified to initiate the survey (task approval rating > 97%, successful completion of more than 100, but fewer than 10,000 tasks, US-based IP address) [50]. Additional checks were embedded within the survey to screen out potential use of virtual private networks (VPNs) to mimic US-based IP addresses, eliminate bots, and manage careless responses [51]. Failing at these checkpoints resulted in immediate termination of the task and exclusion from the study, but no other exclusion criteria were applied. Participants who successfully completed the survey were compensated $1.00 USD.

### Instrument

#### Sociodemographic questions

Participants were asked to indicate their age (in years), gender [male, female, nonbinary, transgender], race [White, Black or African American, American Indian or Alaska Native, Asian, Native Hawaiian or Pacific Islander, Other], ethnicity [Hispanic or Latino/a], and education level [less than high school, high school or GED, associate’s degree, bachelor’s degree, master’s degree, doctoral or professional degree]. Due to cell sizes, race and ethnicity were merged into a single race/ethnicity variable: [non-Hispanic White, non-Hispanic Black or African American, Hispanic or Latino/a, Asian, and Other].

#### Believability of COVID-19 narratives

Participants were asked to rate the believability of different statements about COVID-19 using a Likert-type scale from 1 (Extremely unbelievable) to 7 (Extremely believable). This response structure was drawn from prior research on believability (e.g., Herzberg et al. [52]).

Four narrative statements were drawn and synthesized from Cornell University’s Alliance for Science [23]. An additional statement was based on the zoonotic explanation [28, 29]. The statements were prefaced with a single prompt, reading: “There is a lot of information available right now about the origins of the COVID-19 virus. We are interested in learning how *believable* you find the following explanations of COVID-19.”

The statements below were used to form the profiles of believability of COVID-19 narratives:

1. “The recent rollout of 5G cellphone networks caused the spread of COVID-19.”
2. “The COVID-19 virus originated in animals (like bats) and spread to humans.”
3. “Bill Gates caused (or helped cause) the spread of COVID-19 in order to expand his vaccination programs.”
4. “COVID-19 was developed as a military weapon (by China, the United States, or some other country).”
5. “COVID-19 is no more dangerous than the flu, but the risks have been exaggerated as a way to restrict liberties in the United States.”

#### Trust in science and scientists

Participants were asked to complete the Trust in Science and Scientist Inventory consisting of 21 questions with 5-point Likert-type response scales ranging from 1 (Strongly disagree) to 5 (Strongly agree). After adjusting for reverse-coded items, the mean value of the summed scores of 21 questions was used to indicate a level of trust ranging from 1 (Low Trust) to 5 (High Trust) [45]. The scale demonstrated excellent reliability for this sample (*α* = .931).

#### Religious commitment

Participants were asked to describe their “level of religious commitment (*this refers to any belief system*)” on a scale from 1 (Low) to 10 (High).

#### Political orientation

Participants were asked to describe their “political orientation” on a scale from 1 (Liberal) to 10 (Conservative).

### Statistical analysis

Four stages of analyses were conducted. First, descriptive statistics were computed and reported for believability of COVID-19 narratives, religious commitment, political orientation, trust in science, and sociodemographic characteristics (e.g., race/ethnicity, sex, sexual orientation, education level). Means and standard deviations (SD) were used to describe continuous variables (e.g., believability of COVID-19 narratives, age). Unweighted frequencies and weighted percentages were used to describe categorical variables (e.g., race/ethnicity, gender). We used Stata 15.1 for statistical description and bivariate inference (Chi-square [*χ*^2^] and *t-*tests).

Second*,* Latent Profile Analysis (LPA) was conducted using Mplus version 8 (Muthen & Muthen, Los Angeles, CA) to delineate subgroups of belief patterns related to COVID-19 among participants [44]. We used maximum likelihood and a robust estimator (Huber-White, MLR estimator in Mplus) to handle the non-normal distribution of the indicators (absolute value of skew ranged from 0.30 to 1.67, and of kurtosis ranged from 1.70 to 4.39). LPA is an unsupervised machine learning technique to identify unobserved groups or patterns from the observed data [44, 53]. Compared to traditional cluster analysis, LPA adapts a person-centered approach to identify the classes of participants who may follow different patterns of beliefs in COVID-19 narratives with unique estimates of variances and covariate influences. Since no other study has investigated this question or these variables, we followed an exploratory approach to identifying the number of classes by testing increasingly more classes until the value of the log likelihood began to level off (1–5 latent classes).

To determine the final number of classes, we systematically considered conceptual meaning [54], statistical model fit indices [55], entropy [56], and the smallest estimated class proportions [55]. Model fit indices included the Akaike Information Criterion (AIC), Bayesian Information Criterion (BIC), and adjusted BIC, with the smaller values representing greater model fit [55, 57–59]. Entropy ranged from 0 to 1, with the higher values indicating better distinctions between the classified groups and a value of 0.60 indicating good separation [60]. Models that included class sizes with less than 1% of the sample or that did not converge were not considered due to the risk of poor generalizability [61]. The Vuong-Lo-Mendel-Rubin Likelihood Ratio Test (LMR) [62] was further used to test whether models with *k* classes improved the model fit versus models with *k-1* classes (a significant *p-value*<.05 suggested such improvement). Full information maximum likelihood (FIML) estimation was used to handle missing data [63–65].

Third, bivariate analyses were conducted between the study variables and the classified groups using analysis of variance (ANOVA). A Bonferroni correction for multiple comparisons was applied. Finally, multivariate multinomial logistic regressions were used to examine the utility of trust in science in identifying COVID-19 narrative groups, adjusting for all sociodemographic variables, political orientation, and religious commitment. Significance testing was 2-sided and carried out at the 5% significance level.

## Results

### Descriptive statistics

Of the 660 participants (see Table 1), 61.82% were male (*n*=408). The majority were White (*n*=399, 60.45%), followed by Hispanic (*n*=121, 18.33%) and Black or African American (*n*=68, 10.3%) participants. The average age of participants was 24.80 (standard deviation [SD] = 11.94). More than half held a bachelor’s degree (*n*=335, 50.83%). The mean scores of political orientation, religious commitment, and trust in science were 4.82 (SD=3.13), 4.82 (SD=3.78) and 3.65 (SD=0.71), respectively.

**Table 1:** Descriptive statistics

	Total Sample (*n=*660)
	*n* (%)/mean (SD)
Sex	
Male	408 (61.82)
Female	246 (37.27)
Non-Binary/Transgender	6 (0.90)
Race/ethnicity	
White	399 (60.45)
Black	68 (10.30)
Hispanic	121 (18.33)
Asian	57 (8.64)
Others	15 (2.27)
Age	24.80 (11.94)
Education Level^a^	
Less than High School	1 (0.15)
High School or GED	109 (16.54)
Associate Degree	72 (10.93)
Bachelor	335 (50.83)
Master	122 (18.51)
Doctoral /Professional	20 (3.03)
Political orientation	4.81 (3.13)
Religious commitment	4.82 (3.78)
Trust in science	3.65 (0.71)
Believability of COVID-19 Narratives	
5G Narrative	1.94 (1.72)
Zoonotic Narrative	5.56 (1.64)
Gates Vaccine Narrative	2.27 (1.88)
Laboratory Narrative	3.28 (2.00)
Liberty Restriction Narrative	2.96 (2.04)

*GED* General Education Development Test or General Education Diploma, *SD* Standard deviation

^a^ Education level was treated as a continuous variable in later analyses

For the full sample, believability of the narratives varied, from a low of 1.94 (SD=1.72) for the 5G narrative to a high of 5.56 (SD=1.64) for the zoonotic narrative. Means for each narrative statement are provided in Table 1.

### Profiles of beliefs in COVID-19 narratives

Based on model fit statistics (see Table 2), we selected a 4-class model. The LMR test was non-significant when comparing the 5-class to the 4-class model, the model fit indices of the 4-class model were the smallest among 1- to 4- class models, and the entropy was over 0.60 and the highest of all the estimated models (entropy = 0.994). The smallest class of the 4-class model was also larger than 5% of the total sample (8.18%).

**Table 2:** Latent profile analysis model fit summary

Model	Log Likelihood	AIC	BIC	SABIC	Entropy	Smallest Class %	LMR *p-value*	LMR meaning
1	− 6682.848	13,385.696	13,430.619	13,398.868			–	–
2	− 5908.942	11,849.884	11,921.759	11,870.959	0.987	0.19091	< 0.001	2> 1
3	− 5707.847	11,459.694	11,558.523	11,488.673	0.989	0.09545	< 0.001	3> 2
4	− 5554.458	11,164.915	11,290.698	11,201.797	0.994	0.08182	< 0.001	4> 3
5	− 5442.434	10,952.868	11,105.604	10,997.653	0.980	0.02424	0.139	5< 4

*n* = 660; The LMR test compares the current model to a model with k −1 profiles

*AIC* Akaike’s Information Criterion, *BIC* Bayesian Information Criterion, *SABIC* Sample-Adjusted BIC, *LMR* Lo-Mendell Ruben

**Fig. 1:**
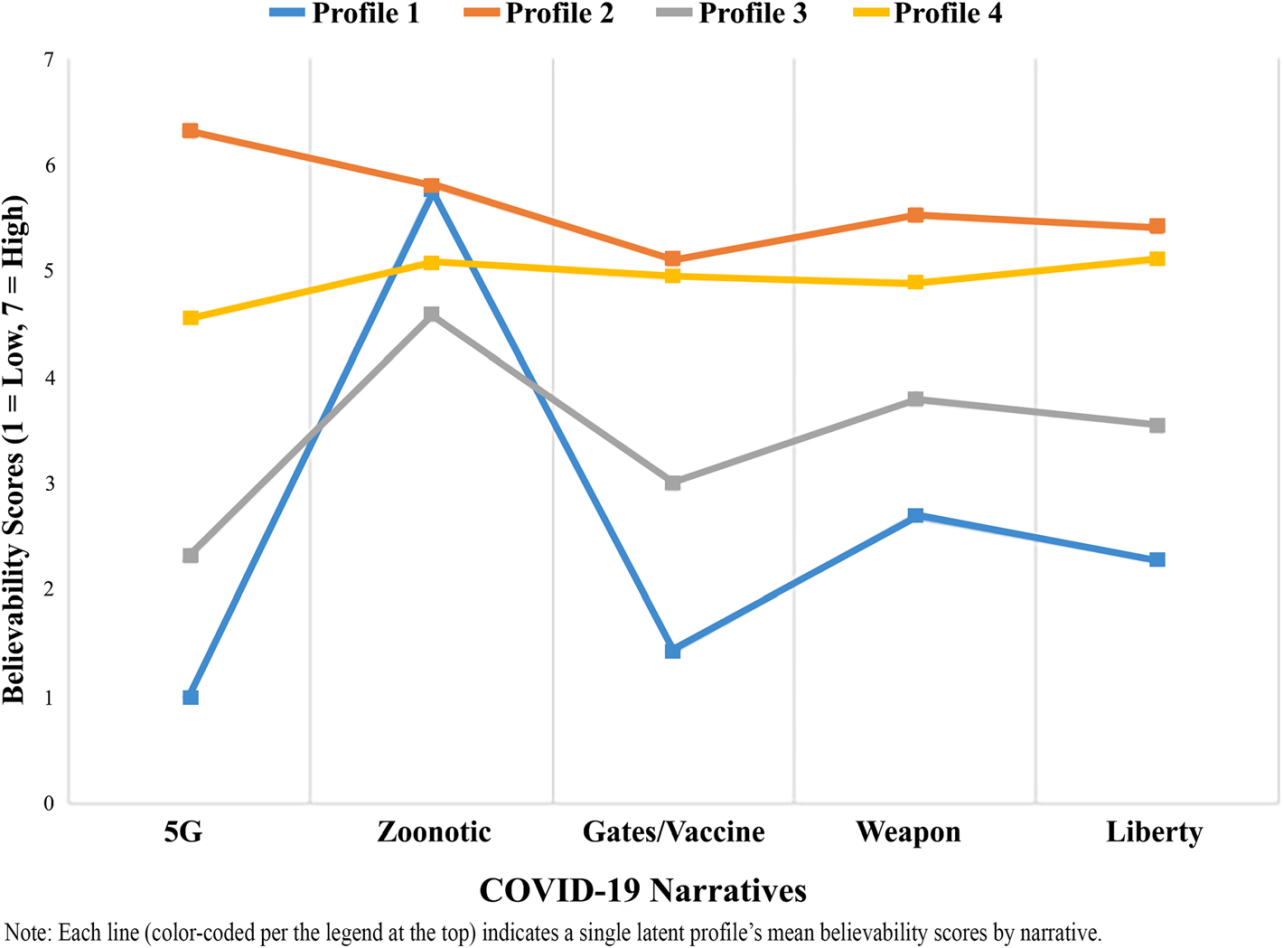
Latent profiles of believability of COVID-19 narratives

Figure 1 shows the mean believability of COVID-19 narratives statements across the 4 identified profiles.

- Profile 1 (*n*= 463, 70.15%), the largest class, generally believed the scientific consensus narrative about COVID-19 and tended not to believe in other narratives. This group reported the lowest believability scores for the 5G narrative (mean = 1.00, SD=0.00), the Bill Gates vaccine narrative (mean = 1.43, SD=1.06), the laboratory development narrative (mean = 2.70, SD=1.83), and the liberty restriction narrative (mean = 2.28, SD=1.67). It also reported high believability for the zoonotic narrative (mean = 5.76, SD=1.61).
- Profile 2 (*n*= 54, 8.18%) considered all of the narrative statements to be highly plausible, reporting the highest believability scores for the 5G narrative (mean = 6.31, SD=0.47), zoonotic narrative (mean = 5.80, SD=1.18), Bill Gates vaccine narrative (mean = 5.11, SD=1.73), laboratory development narrative (mean = 5.52, SD=1.61), and the liberty restriction narrative (mean = 5.41, SD=1.78).
- Profile 3 (*n*= 77, 11.67%) reported low-to-moderate believability for all of the narrative statements. In most cases, this class had the second-lowest belief scores for narrative statements, but also, notably, the lowest score for the zoonotic narrative (mean = 4.59, SD=1.71).
- Profile 4 (*n*= 66, 10.00%) reported fairly high believability for most narratives (similarly to Profile 2). However, this group diverged from Profile 2 in indicating lower plausibility of the 5G narrative (mean = 4.55, SD=0.50), though it was still a higher level of belief than for Profiles 1 and 3.

As indicated in Table 3, profiles differed significantly across racial/ethnic groups, education levels, political orientation, religious commitment, and trust in science. These findings are provided for transparency and context, but the primary associative findings are those in the next subsection (e.g., the multivariate models).

**Table 3:** Descriptive statistics by four latent profiles

Variables	Profile 1	Profile 2	Profile 3	Profile 4
	(*n*= 463, 70.15%)	(*n*= 54, 8.18%)	(*n*= 77, 11.67%)	(*n*= 66, 10.00%)
	n (%)/mean (SD)	n (%)/mean (SD)	n (%)/mean (SD)	n (%)/mean (SD)
**Sex**				
Male	274 (59.18)	41 (75.93)	49 (63.64)	44 (66.67)
Female	183 (39.52)	13 (24.07)	28 (36.36)	22 (33.33)
Binary/Trans	6 (1.30)	0 (0.00)	0 (0.00)	0 (0.00)
Race/ethnicity***				
White	326 (70.41)	14 (25.93)	38 (49.35)	21 (31.82)
Black	29 (6.26)	11 (20.37)	16 (20.78)	12 (18.18)
Hispanic	47 (10.15)	27 (50.00)	16 (20.78)	31 (46.97)
Asian	48 (10.37)	2 (3.70)	5 (6.49)	2 (3.03)
Others	13 (2.81)	0 (0.00)	2 (2.60)	0 (0.00)
**Age**	24.89 (12.02)	23.98 (11.62)	25.17 (11.91)	24.42 (11.88)
Education level***	3.70 (1.06)	4.17 (0.93)	3.83 (0.86)	4.17 (0.90)
Political orientation***	4.17 (3.00)	6.96 (2.81)	5.22 (2.80)	7.08 (2.74)
Religious commitment***	3.84 (3.82)	7.56 (2.44)	6.43 (2.73)	7.55 (1.92)
Trust in science***	3.90 (0.64)	2.92 (0.42)	3.27 (0.56)	2.93 (0.38)
**Believability of COVID-19 Narratives**				
5G Narrative	1.00 (0.00)	6.31 (0.47)	2.32 (0.47)	4.55 (0.50)
Zoonotic Narrative	5.76 (1.61)	5.80 (1.18)	4.59 (1.71)	5.08 (1.58)
Gates Vaccine Narrative	1.43 (1.06)	5.11 (1.73)	3.01 (1.56)	4.95 (1.62)
Laboratory Narrative	2.70 (1.83)	5.52 (1.61)	3.79 (1.59)	4.89 (1.49)
Liberty Restriction Narrative	2.28 (1.67)	5.41 (1.78)	3.55 (1.78)	5.11 (1.66)

*n* = 660

*SD* Standard deviation

### Multivariate models predicting COVID-19 belief profiles

The multivariate regression models (see Table 4) contrasted Profiles 2 through 4 with Profile 1 (which was the profile expressing belief in the zoonotic narrative but the lowest belief in the other narratives). Controlling for race/ethnicity, gender, age, and education level, individuals with greater trust in science were less likely to be in Profile 2 (AOR=0.07, 95%CI=0.03–0.16), Profile 3 (AOR=0.20, 95%CI=0.12–0.33), and Profile 4 (AOR=0.07, 95%CI=0.03–0.15) than Profile 1. In addition, study participants with greater religious commitment were more likely to be in Profile 3 (AOR=1.12, 95% CI = 1.02–1.22) than Profile 1. No other significant differences related to religious commitment were observed, though it appears that with a larger sample size a similar religious effect may have been significant for Profiles 2 and 4. Political orientation was not associated with belief profiles in the multivariate models.

**Table 4:** Multivariate multinomial logistic regressions (reference = profile 1)

Variables	Profile 2	Profile 3	Profile 4
	(*n*= 54, 8.18%)	(*n*= 77, 11.67%)	(*n*= 66, 10.00%)
	AOR (95%CI)	AOR (95%CI)	AOR (95%CI)
Political orientation	0.98 (0.85–1.13)	0.90 (0.81–1.00)	1.01 (0.88–1.15)
Religious commitment	1.16 (0.99–1.34)	1.12 (1.02–1.22)*	1.14 (1.00–1.31)
Trust in science	0.07 (0.03–0.16)**	0.20 (0.12–0.33)**	0.07 (0.03–0.15)**

*AOR* Adjusted Odds Ratio, *95% CI* 95% Confidence Interval; Controlled for race/ethnicity, gender, age, and educational levels

*p< 0.05 **p< 0.001

## Discussion

This study tested two preliminary hypotheses about beliefs in narratives about COVID-19. We had hypothesized that individuals would be separable into distinct latent classes based on belief in various narratives about COVID-19, and the LPA analysis identified four statistically and conceptually different subgroups. Further, we speculated that trust in science was lower among that groups that reported high believability for misinformation about COVID-19, which was partially supported by our results. These results should be interpreted as supporting the plausibility of these explanations, but as always, should be replicated and further investigated before definitive conclusions are made. We specifically encourage further replication and extensions of this work and support open dialogue about the findings and their implications.

### Profiles of COVID-19 belief subgroups

Prior research on conspiracy theories has suggested that many people in the US believe in at least one conspiracy theory [12], and that those who do may believe in multiple conspiracy theories [13]. Our LPA analysis, which included believability not only of conspiracy theories/misinformation, but also of the current scientifically-accepted zoonotic explanation for COVID-19, affirmed this finding and added considerable detail.

Profile 1 reported the lowest believability for each misinformed narrative and reported high believability of the zoonotic narrative. This may suggest that people who are skeptical of misinformation tend to believe the scientifically accepted narrative. Interestingly, however, the converse was not true. In fact, the highest believability in the zoonotic explanation was observed for Profile 2, which reported the highest believability for all explanations. Further, Profile 4 was fairly similar to Profile 2, except for lower endorsement of the 5G theory, which we subjectively note is the least plausible theory on its face, given a complete lack of scientific evidence that wireless technology can transmit a virus. Finally, Profile 3 reported low to moderate believability for all narrative statements but reported the lowest endorsement for the zoonotic explanation. This is also important to note, as it suggests that a generally neutral position on the believability of misinformed narratives does not necessarily translate to endorsement of a scientifically-accepted narrative.

Our data support the existence of multiple and distinct belief profiles for COVID-19 misinformation. Based on these findings, we speculate that one reason providing factual information has not always reduced endorsement of misinformation [41] is that latent groups of people exist for whom belief in a scientifically-accepted explanation is not a mutually exclusive alternative to belief in misinformation (e.g., Profiles 2 and 4). For people belonging to these subgroups, convincing them of the validity of the scientifically-accepted explanation may simply increase their belief in that explanation, without concomitant reductions in belief in alternative narratives. In addition, it is important to note that even Profile 1, which was the most skeptical of misinformation and which expressed high believability for the zoonotic explanation, reported a mean believability value > 2 for two alternative narratives (laboratory development and liberty restriction). Though such narratives are not strongly supported by currently-available evidence, neither are they scientifically impossible (as is the 5G theory). The liberty restriction narrative, in particular, is multifaceted. While evidence continues to accumulate that COVID-19 is a more serious health threat than influenza (e.g., US Centers for Disease Control and Prevention provisional death counts [66]), there may still be disagreement about the appropriate public health response. For example, even given the evidence for substantial and positive outcomes from mask-wearing requirements [38], their implementation continues to be contentious. Thus, in some ways, failure to reject all alternative narratives with complete certainty better reflects true scientific work better than would absolute rejection of all alternative narratives [40], because they may reflect complex and interlinked systems of beliefs.

### Predictors of COVID-19 belief subgroups

In our multinomial logistic regression models, controlling for race/ethnicity, gender, age, and education level (as well as the other predictor variables), political orientation was not significantly associated with belonging to any particular COVID-19 belief subgroup. This finding is consistent with some prior hypotheses [12], but it is important to reiterate, given the tenor of current political discussion in the US. This is not to say that a bivariate or multivariate association between belief in misinformation and political orientation cannot be identified [67], but it is to suggest the possibility that trust in science may be an underlying variable driving this differentiation.

Although religious commitment was significantly associated with being part of Profile 3 versus Profile 1, the magnitude of this association was not particularly large in comparison to the findings related to trust in science. In addition, examining the confidence intervals independently of significance levels, one might reasonably speculate that belonging to any of Profiles 2 through 4 might be potentially associated with increased religious commitment. It may be the case that the trust in science variable captures some of the complexity that has been observed in associating religion and belief in misinformation [22].

Finally, low trust in science was substantially and significantly predictive of belonging to Profiles 2, 3, and 4, relative to Profile 1. However, those profiles were distinguished from Profile 1 *not* by their failure to believe in the zoonotic explanation, but by their endorsement of alternate explanations. In other words, trusting science and scientists appears to be associated with lower likelihood of expressing a belief pattern that endorses narratives that are definitively, or likely to be, misinformed. In this sense, trust in science was conceptually less related to what narrative to believe, and more related to what narrative(s) are more appropriate to disbelieve.

It is important, on a surface level, to understand the potential importance that trust in science has in understanding how people perceive competing narrative explanations about a major event like the COVID-19 pandemic. Unlike political orientation and religious commitment, which can become part of a personal identity (and hence may be more difficult to modify), trust in science is, on its face, a potentially modifiable characteristic. From a public health standpoint, the strength of the association between trust in science and misinformation believability profiles, combined with the potential mutability of the ‘trust in science’ variable, may indicate a potential opportunity for a misinformation intervention. However, the solution is not likely to be as simply as “just asserting that science can be trusted.” First, consider the conflict described earlier in this manuscript, where there is an inherent tension between conspiratorial thinking and trusting expert opinion. If it were true, for example, that 5G networks were being used to spread COVID-19, then the authorities doing so, and desiring to hide it, would have an interest in debunking the 5G narrative. If “science” and “authority” or “government bodies” become conflated, then lower trust in science may result from distrust of authority, thereby affecting believability of explanations [68]. Thus, one important consideration might be the importance of working to ensure that science remains non-partisan, including careful vigilance for white hat bias (distortion of findings to support the “correct” outcome) [69].

Second, although as researchers we believe in the power of the scientific approach to uncover knowledge, there have been well-documented cases of scientific misconduct, such as the 1998 Wakefield et al. paper linking vaccines and autism [70], as well as other concerns about adherence to high-integrity research procedures [71]. Anomalies or other issues related to research partnerships can occur as well. While this paper was being prepared for submission, a major COVID-19 study on hydroxychloroquine was retracted due to issues with data access for replication [72]. At the same time, as researchers, we understand that a single study does not constitute consensus, and that not all methods and approaches yield the same quality of evidence. Science, as a field, scrutinizes itself and tends to be self-correcting – though not always as rapidly as one might wish, and systems regularly have been reconfigured to ensure integrity [73]. In the time between submission and revision of this paper following peer review, randomized, controlled trials of hydroxychloroquine have been published and have served to disambiguate its clinical utility for COVID-19 (e.g., the RECOVERY trial) [74]. In this case, the scientific approach appears to have functioned as intended – over time. However, to a person not embedded within the scientific research infrastructure, it is not necessarily irrational to report a lower level of trust in science on the basis of the idea that certain scientific theories have been wrong, study findings do not always agree, and in rare cases, findings have been fraudulently obtained.

Given that trust in science and scientists was the most meaningful factor predicting profile membership, accounting for a wide variety of potential covariates, systematically building trust in science and scientists might be an effective way to inoculate populations against misinformation related to COVID-19, and potentially other misinformation. Based on this study’s findings, this would specifically not take the form of repeatedly articulating factual explanations (especially within a scientific echo chamber [43]), as this might potentially increase believability of accurate narratives, but only as one among other equally believable narratives. Rather, to improve trust in science, we might consider demonstrating – honestly and openly – how science works, and then articulating why it can be trusted [40]. Parallel processes such as implementing recommendations to facilitate open science [75] may also have the secondary effect of improving overall public trust in science. Individuals who both understand [20, 21] and trust science [7, 45] appear to be most likely to reject explanations with less supporting evidence while accepting narratives with more supporting evidence.

### Limitations

This study has several limitations. First, to conduct rapid research amid a pandemic, we used the mTurk survey platform. As noted in our Methods, this is a widely accepted research platform across multiple disciplines, but it does not produce nationally representative data. Thus, the findings should not be generalized to any specific population without further study. In addition, we suspect, but cannot confirm, that the results would potentially look different outside of the US. Second, because COVID-19 emerged recently, and research on COVID-19 misinformation was initiated even more recently, no validated questionnaires for believability of COVID-19 misinformation existed at the time of survey administration. However, we suggest some face validity for our measures of misinformation believability because the response scale was established in prior research [52] and because the topics were drawn from a reputable list of misinformed narratives [23]. Third, as with all inferential models, this study is subject to omitted variable bias [76], though the magnitude of the association between the latent profiles and the trust in science variable somewhat attenuates this concern. Fourth, since this was a cross-sectional study, we cannot assert any causality or directionality.

## Conclusions

Misinformation related to COVID-19 is prolific, has practical and negative consequences, and is an important area on which to focus research. This study adds to extant knowledge by finding evidence of four differential profiles for believability of COVID-19 narratives among US adults. Those profiles suggest that believing misinformation about COVID-19 may not be mutually exclusive from believing a scientifically accepted explanation, and that most individuals who believe misinformation believe multiple different narratives. Our work also provides provisional evidence that trust in science may be strongly associated with latent profile membership, even in the presence of multiple covariates that have been associated with COVID-19 misinformation in other work (e.g., political orientation).

We propose several next steps after the current work. First, a larger, nationally representative sample of individuals in the US should complete these items, potentially also including common misinformation or conspiracy theories about other topics likely to affect health behaviors, like vaccination [77]. Second, it will be important for future studies to determine whether our study’s findings can be replicated, are highly generalizable, and whether additional nuances to the findings can be identified by the broader scientific community. Further, longitudinal studies could be structured to enable causal inferences from the profiles. Third, there may be utility in validating a general set of measures related to COVID-19 misinformation believability. Finally, randomized experiments to determine whether brief interventions can improve trust in science, and thereby affect latent profile membership – or even preventive behavioral intentions – might be useful in supporting the US public health infrastructure.

## Supplementary Information

**Additional file 1.**

## Data Availability

Raw data and analytic code are uploaded as supplemental files with this article.

## Abbreviations

ANOVA: Analysis of variance
AIC: Akaike information criterion
BIC: Bayesian information criterion
COVID-19: Coronavirus disease 2019
FIML: Full information maximum likelihood
LPA: Latent profile analysis
mTurk: Amazon Mechanical Turk
LMR: Vuong-Lo-Mendel Likelihood Ratio Test
