# Supplementary material for: Misinformation about COVID-19: evidence for differential latent profiles and a strong association with trust in science
Source: BMC Public Health. 2021 Jan 7;21:89. doi: 10.1186/s12889-020-10103-x (PMC7789893; doi:10.1186/s12889-020-10103-x)
Supplement: Supplementary file 1 — Additional file 1. [file 12889_2020_10103_MOESM1_ESM.zip › BMC Public Health 6112020 codeR1.docx]

**Data:**

**#Directory#**

**Variable:**

**Names are**

**Religious Political Education Q18_1 Q18_2 Q18_3 Q18_4 Q18_6 CurrentTrust**

**male female nonbinary trans race white black hispanic asian others**

**Age ;**

**Usevariables are**

**Q18_1 Q18_2 Q18_3 Q18_4 Q18_6 ;**

**! IDVARIABLE is RandomID;**

**Missing are all (99) ;**

**CLASSES = c(4);**

**AUXILIARY = (R3STEP) Religious Political Education**

**CurrentTrust male female nonbinary trans**

**white black hispanic asian others**

**Age ;**

**Analysis:**

**TYPE=MIXTURE ;**

**ESTIMATOR = MLR;**

**STARTS = 1000 250;**

**STITERATIONS = 500;**

**LRTSTARTS = 2 1 50 10;**

**MODEl:**

**%OVERALL%**

**[ Q18_1 Q18_2 Q18_3 Q18_4 Q18_6 ];**

**Q18_1 Q18_2 Q18_3 Q18_4 Q18_6 (Var1-Var5);**

**OUTPUT: TECH1 TECH4 TECH8 TECH11 TECH14;**

**SAVEDATA:**

**FILE IS LPA_COVID_4.dat;**

**SAVE = bchweights;**

**Plot:**

**type = plot3;**

**series =**

**Q18_1 Q18_2 Q18_3 Q18_4 Q18_6 (*);**
